# Italian multicentre observational study of the prevalence of CCSVI in multiple sclerosis (CoSMo study): rationale, design, and methodology

**DOI:** 10.1007/s10072-012-1269-5

**Published:** 2013-01-24

**Authors:** Giancarlo Comi, Mario Alberto Battaglia, Antonio Bertolotto, Massimo Del Sette, Angelo Ghezzi, Giovanni Malferrari, Marco Salvetti, Maria Pia Sormani, Luigi Tesio, Erwin Stolz, Gianluigi Mancardi

**Affiliations:** 1 Division of Neurology and Neurophysiology Service, Ospedale “San Raffaele”, Milan, Italy; 2 Fondazione Italiana Sclerosi Multipla, Via Operai 40, 16149 Genoa, Italy; 3 Multiple Sclerosis Center, Ospedale “San Luigi”, Orbassano, Italy; 4 Neurology Unit, Ospedale “Sant’ Andrea”, La Spezia, Italy; 5 Multiple Sclerosis Research Center, Ospedale “Sant’ Antonio Abate”, Gallarate, Italy; 6 Neurology Unit-Stroke Unit, Department of Neuromotor Physiology, Istituto di Ricovero e Cura a Carattere Scientifico, Arcispedale Santa Maria Nuova, Reggio Emilia, Italy; 7 Center for Experimental Neurological Therapies, Università “La Sapienza”, Rome, Italy; 8 Department of Health Sciences, Università di Genova, Genoa, Italy; 9 Department of Biomedical Sciences for Health, Università degli Studi ed Istituto Auxologico Italiano, Milan, Italy; 10 Department of Neurology, Justus-Liebig-Universität, Giessen, Germany; 11 Department of Neuroscience, Rehabilitation, Ophthalmology, Genetics, Maternal and Child Health, Università di Genova, Genoa, Italy

**Keywords:** Multiple sclerosis, CCSVI, Color-coded duplex sonography, Observational, Multicenter, CoSMo

## Abstract

Chronic cerebro-spinal venous insufficiency (CCSVI) has been proposed as a “congenital malformation” implicated in the pathogenesis of multiple sclerosis (MS). However, numerous studies failed to confirm its presence in MS patients. This paper presents the rationale, design, and methodology adopted in the CoSMo study, conducted with the aim of verifying whether or not CCSVI is linked to MS. The primary endpoint of the CoSMo study is to compare the prevalence of CCSVI in patients with MS versus patients affected by other neurodegenerative diseases (OND) and healthy volunteers. CoSMo is a multicenter, blinded, prevalence study recruiting 2,000 adult subjects, involving 43 MS centers across Italy. Assessment of the presence or absence of CCSVI is performed by color-coded duplex (CCD) sonography and two out of the five criteria according to Zamboni are necessary for the diagnosis of CCSVI. Local CCD examination carried out by a certified sonologist and the central image readings performed by experts in the field are blinded. An advanced protocol is also described in this paper. The application of a rigorous methodological design will definitively confirm whether an association exists between CCSVI and MS. Should an association be observed, this study also further examines the link between CCSVI and the severity of MS. The addition of subgroups without MS and OND also provides information on whether CCSVI is specific to MS only. Results from the CoSMo study will play a crucial role in the possible studies concerning the potential treatment of CCSVI in MS.

## Background

Multiple sclerosis (MS) is an inflammatory demyelinating disease of the central nervous system (CNS) resulting from a complex combination of genetic and environmental factors [1]. Evidence of the immunological pathogenesis of the disease is derived from animal models of the disease, the experimental allergic encephalomyelitis, and from the efficacy of treatments targeting the presumed immunological dysfunctions at different levels [2, 3]. However, in recent years, a new potential mechanism has been proposed to contribute to the pathogenesis of MS: the presence of presumably abnormal venous hemodynamics leading to cerebral venous congestion. This condition has been named “chronic cerebro-spinal venous insufficiency” (CCSVI). It is hypothesized that extracranial venous obstruction may lead to inadequate cerebral drainage, raising the venous pressure and stretching the vein walls sufficiently to separate the tight junctions between the endothelial cells that form the blood–brain barrier. Colloids and erythrocytes may then pass through the exposed porous basement membranes and participate in the inflammatory process [4]. CCSVI has been initially reported to be strongly associated with MS [5, 6]. Several subsequent studies have been performed with the aim of reproducing Zamboni’s results, with contradicting outcomes. While some studies confirm Zamboni’s findings [7–11], others claim to have found compelling evidence against a significant contribution of CCSVI to the pathogenesis of MS [12–16]. A systematic review of CCSVI findings in MS has been compiled by Thapar and coworkers [17], who eloquently concluded that “there is substantial variation in the strength of association between CCSVI and MS beyond that explained by demographic differences or sonographer training. Reliable evidence on which to base decisions requires sonographic consensus and assessment of the reproducibility of individual criteria between trained sonographers” [17]. This controversial problem, therefore, clearly needs to be addressed by adopting the highest possible scientific standards and thereby bypassing all limitations inherent in previous studies, such as the limited number of patients [4, 5, 7, 9, 11–13, 15, 16, 18], lack of blinding [5, 7, 8, 10, 11, 13–16], lack of appropriate controls [7–13, 15, 18], lack of multicentric design [4, 5, 7–16, 18], and lack of inter-observer variability assessment [4, 5, 7, 9–16, 18]. The aim of the CoSMo study, named after the Italian “CCSVI: studio Osservazionale Sclerosi Multipla e OND”, that is “Observational Study of the prevalence of CCSVI in Multiple sclerosis and in other neurodegenerative diseases (OND)”, is to conclude the heated debate that has arisen in recent years and provide an appropriate answer as to whether or not CCSVI is associated to MS. This study evaluates the prevalence of CCSVI and of the irregularities of the extracranial cerebro-spinal veins in patients with different MS courses as well as in patients with OND of different origin such as degenerative, vascular inflammatory, and autoimmune of the central and peripheral nervous system, and compare it with the prevalence of CCSVI in healthy controls. The addition of these control subjects and non-MS disorders will help determine whether CCSVI is specific to MS or not. Promoted by the non-profit organization FISM (Fondazione Italiana Sclerosi Multipla, Italian Multiple sclerosis Foundation), this is a multicentric, observational study which adopts Echo Color Doppler (ECD) examinations performed in a blinded manner, and centralized readings for remote visualization of the radiological images using a network of special devices. The large sample size (estimated 2,000 enrolled patients, 1,200 with MS, 400 with OND and 400 healthy controls), the blinding procedures, the multicentric design, and the validation of the diagnostic criteria are what distinguish this study from those previously performed.

## Methods/design

### Study population

Subjects included in the study population are aged between 18 and 55 years. Three patient groups are included in this study. The first group (MS group) includes patients diagnosed with MS, either with relapsing remitting (RR), secondary progressive (SP), or primary progressive (PP) course, according to McDonald’s criteria and subsequent updates [19, 20] with disease duration between 1 month and 25 years before screening visit and patients with clinically isolated syndrome (CIS) with disease duration of maximum 5 years. Patients must not be in clinical relapse of the disease (at least 30 days since the last relapse). The second group includes healthy controls (HC group), namely subjects without any relevant disease and without any family history of MS or family relation to another MS patient. The third group includes patients with OND. Two subtypes of patients belong to the OND group: patients with neurodegenerative diseases (ONDn), such as Parkinson’s disease or amyotrophic lateral sclerosis and patients with inflammatory CNS disorders (ONDi), such as neuromyelitis optica (NMO), acute disseminated encephalomyelitis (ADEM), encephalitis, and neurolupus. Exclusion criteria for all groups include the presence of acute or chronic invalidating diseases which could interfere with the design or objective of the study, cardiac dysfunction (NYHA class ≥1), previous episodes of venous thromboembolism, neoplasms, thrombophilia, diabetes, primary or secondary pulmonary hypertension and under treatment for this condition, systemic steroid treatment within the past 30 days, past or present cerebrovascular diseases, episodes of transient global amnesia, pregnancy, and previous diagnosis of/treatment for CCSVI. This study plans to recruit at least 1,200 MS patients, 400 HCs, and 400 patients with OND.

### Study design and duration

This is an observational, case–control, cross sectional, multicentric study, for which 43 Italian MS centers and the related sonologists’ groups, evenly distributed through the Italian territory, are participating. Physicians performing the CCD examination, examiners reading the CCD images, the Contract Research Organization (CRO) collecting all data as well as the statistician performing all analyses, are blinded throughout the whole study. Each centre is invited to recruit 60 MS patients (30 RR, 15 SP, 10 CIS, 5 PP), 20 HC and 20 OND patients, but the recruitment is competitive. The study was undertaken in November 2010 and results are expected in 2 years.

### Procedures

A training course with a final examination of proficiency that certifies that the sonologist can perform ultrasound evaluations according to the sonological protocol of the CoSMo study, in its basic or advanced part, is required by the examiners, in order to increase the homogeneity of the diagnostic procedures. The sonologists are physicians already expert in CCD examination of the arteries of the neck and brain. The training course for the CoSMo study lasts at least 2 months. The educational and training pathway of the CoSMo study has been designed and performed with the contribution of two Italian neurosonology societies, the SINV (Società Italiana Interdisciplinare NeuroVascolare, Italian Interdisciplinary NeuroVascular Society) and the SINSEC (Società Italiana di Neurosonologia ed Emodinamica Cerebrale, Italian Society of Neurosonology and Cerebral Hemodynamics). The two societies cooperated in the organization and management of the theoretical and practical training of the proposed sonologists, identifying six centers tutoring each sonologist during the educational phase. They also arranged the final exam to give the certification for performing the ultrasound examination for the CoSMo study, for the basic or for the advanced protocol, depending on the expertise and level of knowledge of each sonologist.

**Fig. 1:**
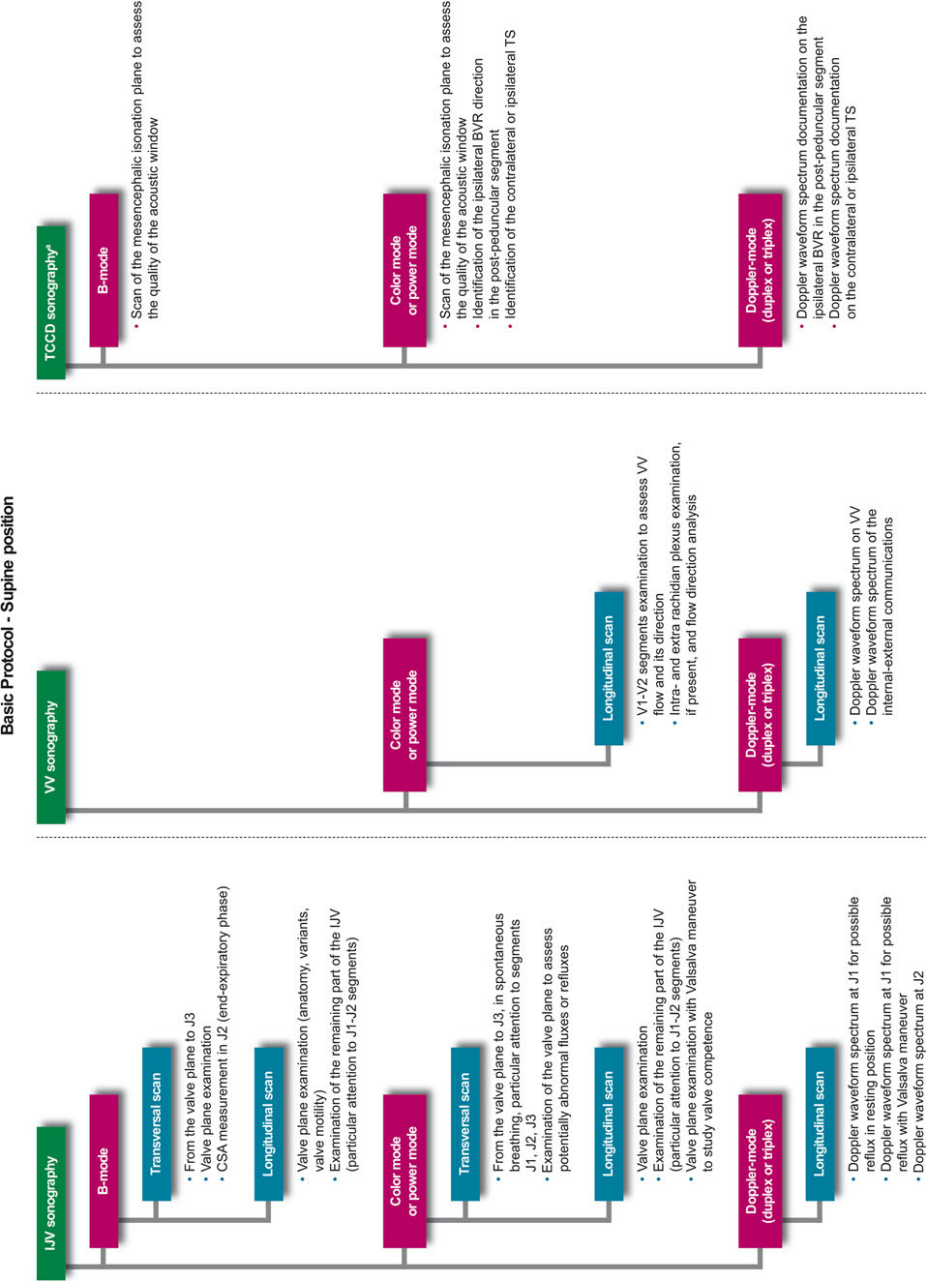
Basic operative protocol for the color-coded duplex sonography in supine position. *IJV* internal jugular vein, *J1, J2, J3* distal (from the valve plane to 0.5 cm from it), middle (at the level of the thyroid gland) and proximal (at the level of the carotid bifurcation) jugular vein segments, *CSA* cross-sectional area, *VV* vertebral veins, *V1, V2* distal and proximal vertebral vein segments, *TCCD* transcranial color-coded duplex sonography, *BVR* basal vein of Rosenthal, *TS* transverse sinus. ^a^ Time window, axial scan, mesencephalic and diencephalic plane; examination has to be performed on both sides, in order to insonate both *right* and *left* temporal windows

**Fig. 2:**
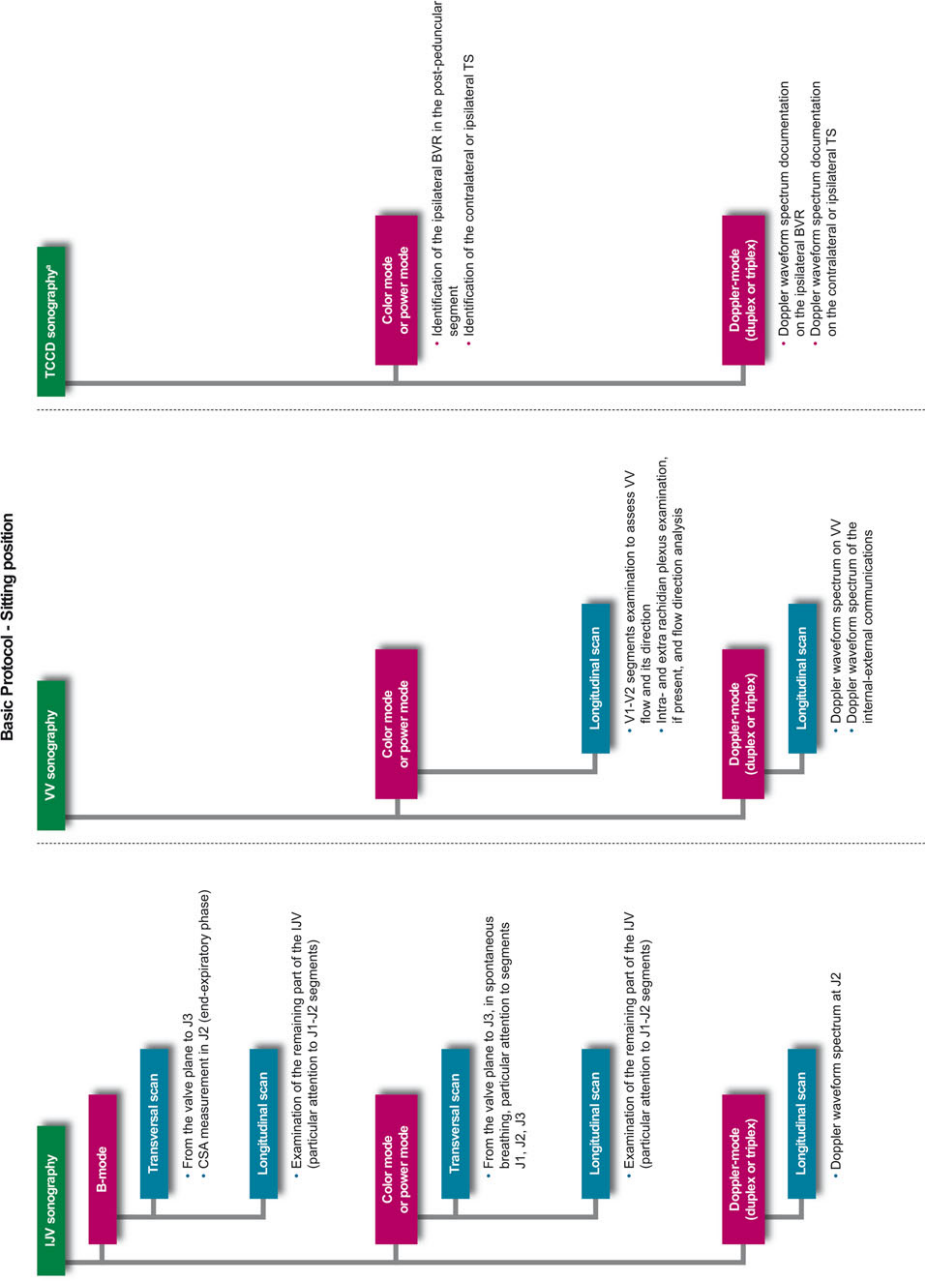
Basic operative protocol for the color-coded duplex sonography in sitting position. *IJV* internal jugular vein, *J1, J2, J3* distal (from the valve plane to 0.5 cm from it), middle (at the level of the thyroid gland) and proximal (at the level of the carotid bifurcation) jugular vein segments, *CSA* cross-sectional area, *VV* vertebral veins, *V1, V2* distal and proximal vertebral vein segments, *TCCD* transcranial color-coded duplex sonography, *BVR* basal vein of Rosenthal, *TS* transverse sinus. ^a^ Time window, axial scan, mesencephalic and diencephalic plane; examination has to be performed on both sides, in order to insonate both *right* and *left* temporal windows

**Fig. 3:**
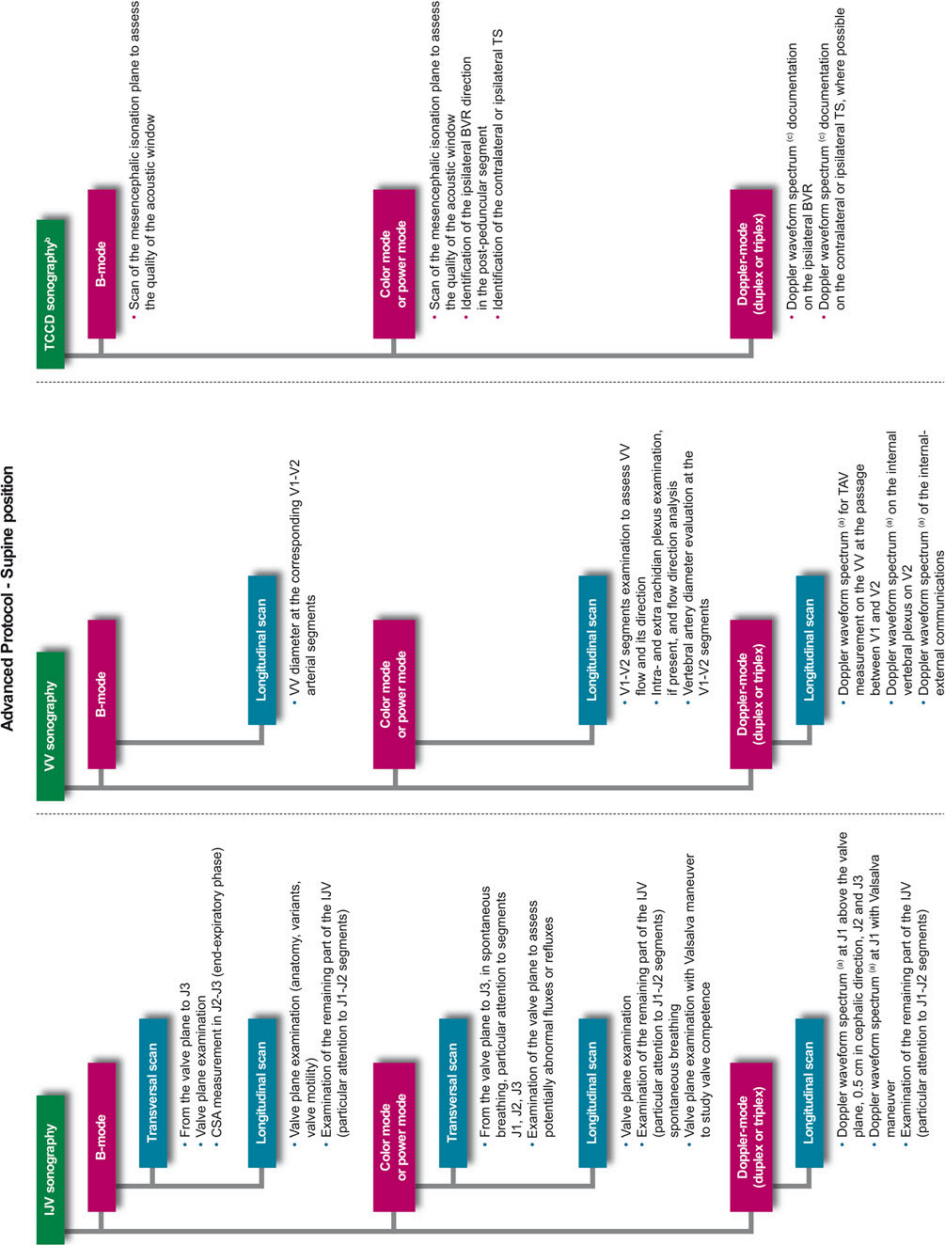
Advanced operative protocol for the color-coded duplex sonography in supine position. *IJV* internal jugular vein, *J1, J2, J3* distal, middle and proximal jugular vein segments, *CSA* cross-sectional area, *VV* vertebral veins, *V1, V2* distal and proximal vertebral vein segments, *TCCD* transcranial color-coded duplex sonography, *BVR* basal vein of Rosenthal, *TS* transverse sinus. ^a^ Doppler waveform spectrum includes *PSV* peak systolic velocity, *EDV* end diastolic velocity and *TAV* time averaged velocity measurements through the automatic or manual selection of at least three cardiac consecutive cycles; blood flow (BF) rate is calculated according to the following formula: BF = CSA × TAV; ^b^ Time window, axial scan, mesencephalic and diencephalic plane; examination has to be performed on both sides, in order to insonate both right and left temporal windows; ^c^ Doppler waveform spectrum includes PSV and EDV measurements

**Fig. 4:**
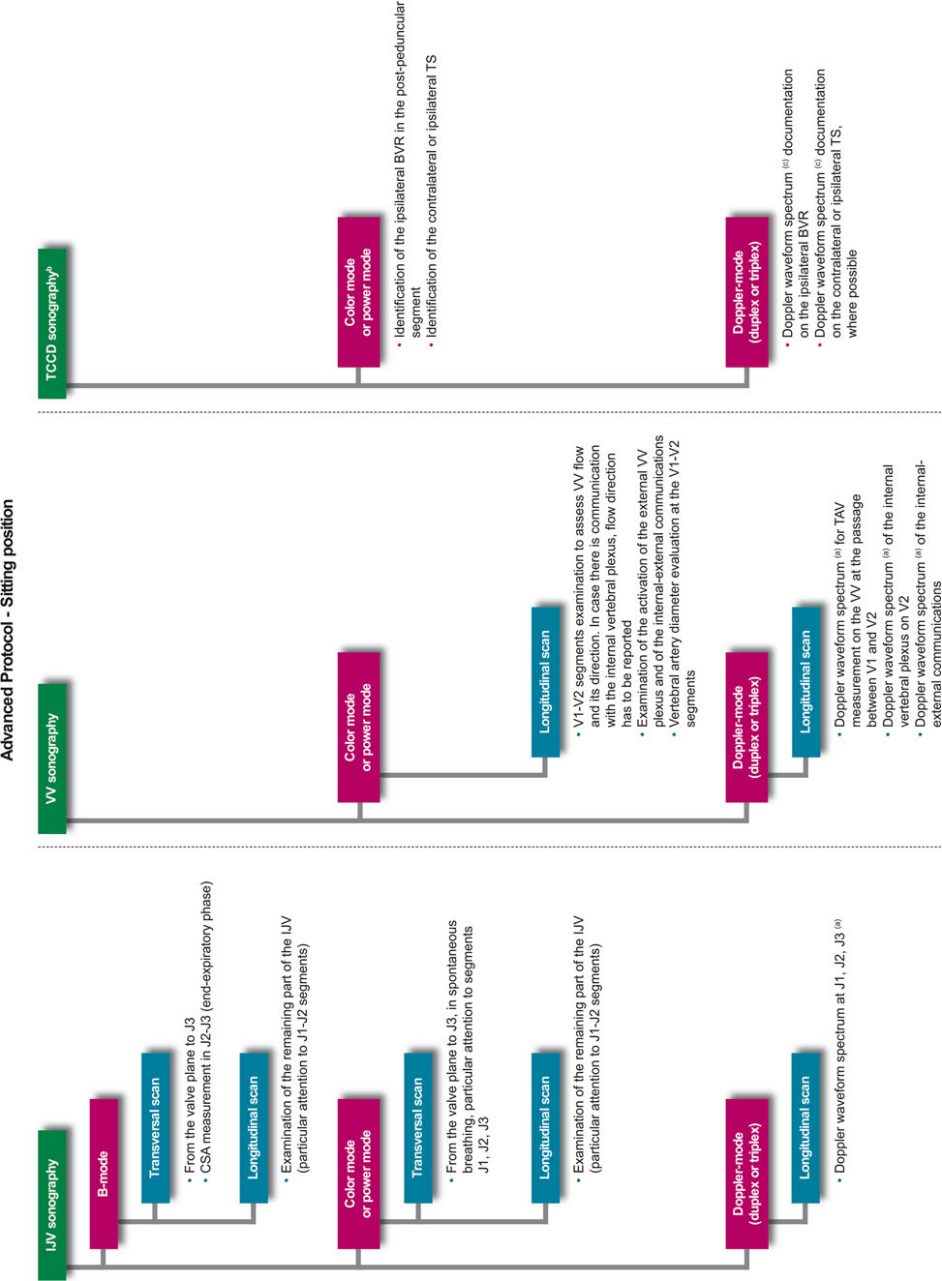
Advanced operative protocol for the color-coded duplex sonography in sitting position. *IJV* internal jugular vein, *J1, J2, J3* distal, middle and proximal jugular vein segments, *CSA* cross-sectional area, *VV* vertebral veins, *V1, V2* distal and proximal vertebral vein segments, *TCCD* transcranial color-coded duplex sonography, *BVR* basal vein of Rosenthal, *TS* transverse sinus. ^a^ Doppler waveform spectrum includes *PSV* peak systolic velocity, *EDV* end diastolic velocity and *TAV* time averaged velocity measurements through the automatic or manual selection of at least 3 cardiac consecutive cycles; blood flow (BF) rate is calculated according to the following formula: BF = CSA × TAV; ^b^ Time window, axial scan, mesencephalic and diencephalic plane; examination has to be performed on both sides, in order to insonate both right and left temporal windows; ^c^ Doppler waveform spectrum includes PSV and EDV measurements

**Fig. 5:**
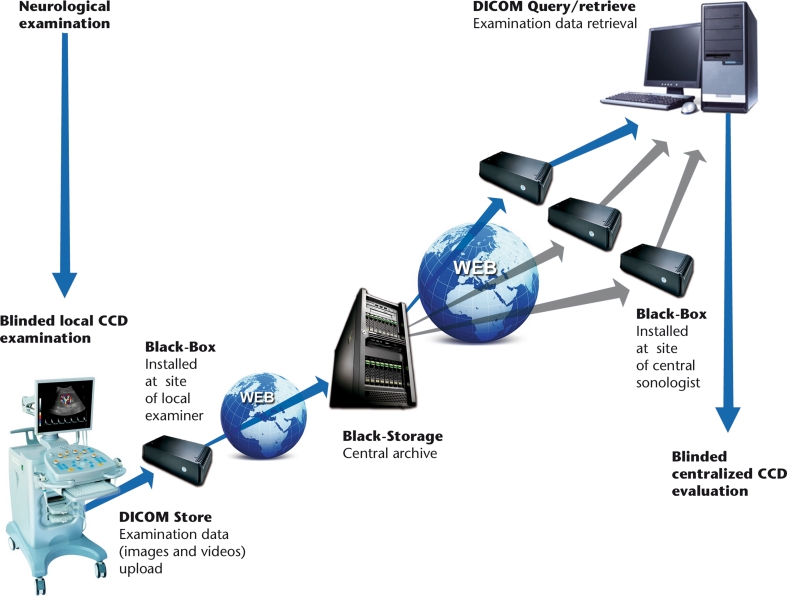
*Black-box* Linkverse^®^ system. Schematic chart describing the flow of images and video recordings from the local examiner performing the CCD examination to the experts performing the centralized readings for CCSVI diagnosis. The* Black-box* Linkverse^®^ uses the universal DICOM format (Digital Imaging and COmmunications in Medicine) and a system for consultation, temporary storage and transmission of images stored in native format and sent as compressed files, without loss of information

Prior to any assessment, an informed consent must be signed by each study participant. For each patient, a screening visit is performed, by a non-blinded neurologist, which provides data on patient’s personal details, medical history, vital signs, and concomitant medications. All patients undergo a physical examination, only MS and OND patients undergo a neurological examination which uses the Barthel Index [21] and, only for MS patients, the expanded disability status score (EDSS) [22]. After the screening visit, the investigator leads the subject to the examiner in charge of performing the CCD blinded examination (CCD Basic protocol; Figs. 1, 2). In order to maintain blinding, subjects are instructed not to communicate with the examiner and are covered to avoid revealing any evidence of medication by injection. The sonologist enters the examination room only after the patient is positioned on the bed. Sonologists who are certified for the advanced protocol, may subsequently perform it in a subgroup of patients, at their discretion. This protocol includes, on top of standard measurements, some additional ones (Figs. 3, 4). The examination is initially performed with the patient in the supine position (Figs. 1, 3) and next in the sitting position (Figs. 2, 4), with a 2 min minimum break between the two positions. At the end of the procedure, the examiner immediately performs his diagnosis (presence or absence of CCSVI) based on the five criteria (described in detail below) and fill the entire online case report form with the required hemodynamic and morphological parameters. The CCD examination images and video recordings are then uploaded for central readings, using the “Black-Box Linkverse^®^” network (Fig. 5). In brief, the CCD evaluation consists in examining the internal jugular veins (IJV) in J1 (distal segment, at the level of the valve plane), J2 (middle segment, at the level of the thyroid gland), and J3 (proximal segment, at the level of the carotid bifurcation); then the vertebral veins (VV) segments V1 (distal segment) and V2 (proximal segment) through axial and longitudinal scans. The mean number of images/video clips per patient is about 100–120. Images are randomly sent to one of three designated central examiners (ES, MDS, GM) who in turn performs a second reading and a diagnosis, blinded to the group affiliation, diagnosis of the local sonologist, and the center. If there is an agreement between the local and the first central examiner a final diagnosis is definitely established. If the two readings have contrasting results, the other two central examiners are asked to read the images and perform a definitive diagnosis. If no consensus is reached, then the diagnosis is accepted according to two out of three central examiners.

### CCSVI diagnostic criteria

In order to diagnose CCSVI, at least two out of the five criteria described in the literature [4] should be met, and the satisfied criteria should be the same as those among the different (local and central) examiners. The criteria are listed below:

1. Constantly present reflux (>0.88 s duration) in internal jugular veins (IJV) and/or vertebral veins (VV) in both seated and supine positions in at least one of the segments J1, J2, J3, V1 or V2, through the color-mode evaluation. Reflux is also defined as flow directed in the vertebral axis, from the extra-rachidian plexus toward the VV (i.e., reflux or reflux on VV and normal or inverted flow on the extra-rachidian plexus).
2. Reflux in the intracranial veins (ICVs, such as the basal vein of Rosenthal or the transverse sinus), analyzed by transcranial color-coded duplex (TCCD) sonography.
3. The presence of anatomical alterations with documented hemodynamic relapses - septa, valve malformations, double channel (one without flow), cross-sectional area (CSA) ≤0.3 cm^2^ - through the B-mode evaluation and Color-mode examination (also by Valsalva maneuver).
4. The absence of flow in IJVs and/or VVs after numerous deep inspirations in both seated and supine positions in at least one of the reference vein segments, through the Color-mode evaluation.
5. Negative difference in the IJV CSA in the J2 segment, that is the area in the upright position subtracted to the area in the supine position (ΔCSA).

The advanced protocol also includes arterial and venous blood flow volume (BFV) measurements [23–25]. Inflow is analyzed in the following districts: common carotid (J2 segment), internal carotid 1 cm after its origin and between V1 and V2 segments. To calculate outflow, CSA and TAV values are measured on the IJV J2 and J3 segments, while the diameter and time averaged velocity (TAV) is measured in the venous segment between V1 and V2.

### Statistical analysis

#### Sample size calculation

A sample size of 1,200 subjects with MS, 400 HC and 400 subjects with OND guarantees a power of 80 % (at a 5 % level of significance) to detect an odds ratio (OR) of 2 (between MS patients and HC or between MS patients and subjects with OND) assuming a prevalence of CCSVI in the reference group (HC) of 5 %, and an OR of 1.50 assuming a prevalence of CCSVI in the reference group of 30 %.

#### Primary end-points

The primary aim of the CoSMo study is to examine the association between CCSVI and MS. Therefore, the null hypothesis of the CoSMo study is the lack of any association between CCSVI and MS. To reject this null hypothesis, the CCSVI prevalence is compared between MS patients and HC and between MS patients and patients with OND by a Chi square test. The null hypothesis is rejected if both tests are significant at a level of 5 %. A Fisher exact test is used in case of observed low cell frequencies (expected counts of <10). The prevalence of CCSVI is calculated along with its 95 % CI in the three study groups (MS, OND and HC) and the strength of the association is evaluated by ORs and their 95 % CI.

#### Secondary end-points

The prevalence of CCSVI is evaluated in CIS, RR, PP, and SP subgroups of MS patients, by Chi square test for heterogeneity and Chi square test for trend.

The impact of other factors such as age, local examiner, and type of scanner on CCSVI, is evaluated by logistic regression analysis. Differences among study groups (MS, HC, and OND) is evaluated by the same model, adjusting for the aforementioned parameters. The same analyses described for CCSVI diagnosis are run for each of the five criteria for CCSVI (prevalence of each criterion, differences among disease groups, adjusted analysis). Inter-rater agreement in CCSVI diagnosis and in each criterion is evaluated between the local and central reading (and among the three central readers when applicable) by Cohen kappa statistic. Also positive and negative agreement will be calculated.

*Trial registration* ClinicalTrials.gov Identifier: NCT01384825.

## Discussion

The very recent claims that CCSVI could be a variable combination of vascular abnormalities playing a role in MS has opened a completely new perspective in the pathogenesis of the disease, with potential therapeutic implications [26, 27]. The topic of CCSVI and MS has gained widespread media attention that is ultimately due to unclear and uncontrolled news. This, in turn, leads to serious discomfort, especially for patients who experience the drama of a pathological condition which has become the subject of biased speculation. Some epidemiological studies, mostly limited by the low number of patients involved, produced quite variable results, from a very strict association between CCSVI and MS [7–11] to the complete absence of association [12–16]. Very soon it became clear that the ultrasound assessment of cerebro-spinal venous abnormalities were highly operator-dependent and influenced by many factors, such as head position, hydration state, respiration, etc. The high level of noise in the ultrasound examination and the subjectivity of the judgment of the examiner must be compensated by a strict training and application of criteria as well as blindness to the diagnosis.

The ultimate aim of the CoSMo study is to respond, with a rigorous study design, to the question as to whether (or not) CCSVI is linked to MS. The stringent methodology adopted, the blinding procedures, the multicentric design, the large sample size, the appropriate controls and the extensive training that has been given to sonologists, are what distinguishes this study from all preceding ones. It is important to underline that only after the demonstration of an association between CCSVI and MS can one consider performing controlled trials to assess the efficacy of interventional treatment as additional therapy for MS. However, a simple association (simultaneous presence of CCSVI and MS) does not equate to causality (CCSVI causing MS).To date, no scientific evidence is available that fulfills the nine causality criteria (strength, consistency, specificity, temporality, biological gradient, plausibility, coherence, experiment, analogy) universally accepted and considered essential by the scientific community to causally correlate a condition and/or a factor with a given disease [28]. Of these nine elements, only “plausibility” and “coherence” could be fulfilled since they require that the alleged cause (CCSVI) is likely to be framed in the context of knowledge and pathogenesis of the disease.

If results from CoSMo support Zamboni’s theory, time and resources can be dedicated to developing or optimizing a cure: this certainly requires controlled studies and it encompasses endovascular techniques and device development to insure durable efficacy of CCSVI surgical treatment.

In order to address some of the controversies on the Zamboni criteria, the CoSMo study also includes an advanced sonological protocol, applied in some centers. These further sonological examinations contribute to a better definition of the intracranial and extracranial pathways of the cerebral venous hemodynamics. This peculiar methodology has been extensively described in a recent publication [29].

## Abbreviations

ADEM: Acute disseminated encephalomyelitis
BFV: Blood flow volume
BVR: Basal vein of Rosenthal
CCSVI: Chronic cerebro-spinal venous insufficiency
CIS: Clinically isolated syndrome
CNS: Central nervous system
CRO: Contract research organization
CSA: Cross-sectional area
DVC: Double check valves
CCD: Color-coded duplex
EDV: End diastolic velocity
FISM: Fondazione Italiana Sclerosi Multipla
HC: Healthy control
IJV: Internal jugular vein
EDSS: Expanded disability status score
MS: Multiple sclerosis
NMO: Neuromyelitis optica
NYHA: New York heart association
OND: Other neurodegenerative disease
ONDi: Other neurodegenerative inflammatory disease
PP: Primary progressive - MS
PSV: Peak systolic velocity
RR: Relapsing remitting - MS
SP: Secondary progressive - MS
TAV: Time averaged velocity
TCCD: Transcranial color-coded duplex sonography
VV: Vertebral veins

## References

[1] Compston A, Coles A (2008). Multiple sclerosis. Lancet.

[2] Chen S-J, Wang Y-L, Fan H-C (2012). Current status of the immunomodulation and immunomediated therapeutic strategies for multiple sclerosis. Clin Dev Immunol.

[3] Fontoura P (2010). Monoclonal antibody therapy in multiple sclerosis: paradigm shifts and emerging challenges. MAbs.

[4] Zamboni P, Galeotti R, Menegatti E (2009). Chronic cerebrospinal venous insufficiency in patients with multiple sclerosis. J Neurol Neurosurg Psychiatr.

[5] Zamboni P, Menegatti E, Weinstock-Guttman B (2009). The severity of chronic cerebrospinal venous insufficiency in patients with multiple sclerosis is related to altered cerebrospinal fluid dynamics. Funct Neurol.

[6] Zamboni P, Consorti G, Galeotti R (2009). Venous collateral circulation of the extracranial cerebrospinal outflow routes. Curr Neurovasc Res.

[7] Al-Omari MH, Rousan LA (2010). Internal jugular vein morphology and hemodynamics in patients with multiple sclerosis. Int Angiol.

[8] Ciccone MM, Galeandro AI, Scicchitano P (2012). Multigate quality Doppler profiles and morphological/hemodynamic alterations in multiple sclerosis patients. Curr Neurovasc Res.

[9] McTaggart RA, Fischbein NJ, Elkins CJ (2012). Extracranial venous drainage patterns in patients with multiple sclerosis and healthy controls. AJNR Am J Neuroradiol.

[10] Zaniewski M, Kostecki J, Kuczmik W (2012). Neck duplex Doppler ultrasound evaluation for assessing chronic cerebrospinal venous insufficiency in multiple sclerosis patients. Phlebology.

[11] Zivadinov R, Poloni GU, Marr K (2011). Decreased brain venous vasculature visibility on susceptibility-weighted imaging venography in patients with multiple sclerosis is related to chronic cerebrospinal venous insufficiency. BMC Neurol.

[12] Mayer CA, Pfeilschifter W, Lorenz MW (2011). The perfect crime? CCSVI not leaving a trace in MS. J Neurol Neurosurg Psychiatr.

[13] Doepp F, Paul F, Valdueza JM (2010). No cerebrocervical venous congestion in patients with multiple sclerosis. Ann Neurol.

[14] Worthington V, Killestein J, Eikelenboom MJ (2010). Normal CSF ferritin levels in MS suggest against etiologic role of chronic venous insufficiency. Neurology.

[15] Centonze D, Floris R, Stefanini M (2011). Proposed chronic cerebrospinal venous insufficiency criteria do not predict multiple sclerosis risk or severity. Ann Neurol.

[16] Baracchini C, Perini P, Causin F (2011). Progressive multiple sclerosis is not associated with chronic cerebrospinal venous insufficiency. Neurology.

[17] Thapar A, Lane T, Nicholas R (2011). Systematic review of sonographic chronic cerebrospinal venous insufficiency findings in multiple sclerosis. Phlebology.

[18] Floris R, Centonze D, Fabiano S (2012). Prevalence study of chronic cerebrospinal venous insufficiency in patients with multiple sclerosis: preliminary data. Radiol Med.

[19] McDonald WI, Compston A, Edan G (2001). Recommended diagnostic criteria for multiple sclerosis: guidelines from the international panel on the diagnosis of multiple sclerosis. Ann Neurol.

[20] Polman CH, Wolinsky JS, Reingold SC (2005). Multiple sclerosis diagnostic criteria: three years later. Mult Scler.

[21] Mahoney FI, Barthel DW (1965). Functional evaluation: the barthel index. Md State Med J.

[22] Kurtzke JF (1983). Rating neurologic impairment in multiple sclerosis: an expanded disability status scale (EDSS). Neurology.

[23] Schreiber SJ, Lurtzing F, Gotze R (2003). Extrajugular pathways of human cerebral venous blood drainage assessed by duplex ultrasound. J Appl Physiol.

[24] Scheel P, Ruge C, Petruch UR, Schöning M (2000). Color duplex measurement of cerebral blood flow volume in healthy adults. Stroke.

[25] Scheel P, Ruge C, Schöning M (2000). Flow velocity and flow volume measurements in the extracranial carotid and vertebral arteries in healthy adults: reference data and the effects of age. Ultrasound Med Biol.

[26] Dake MD (2012). Chronic cerebrospinal venous insufficiency and multiple sclerosis: history and background. Tech Vasc Interv Radiol.

[27] Lugli M, Morelli M, Guerzoni S, Maleti O (2012). The hypothesis of patho-physiological correlation between chronic cerebrospinal venous insufficiency and multiple sclerosis: rationale of treatment. Phlebology.

[28] Hill AB (1965). The environment and disease: association or causation?. Proc R Soc Med.

[29] Malferrari G, Del Sette M, Zedde M (2012). Italian multicenter study on venous hemodynamics in multiple sclerosis: advanced sonological protocol. Perspect Med.

